# Models of HIV Preconception Care and Key Elements Influencing These Services: Findings from Healthcare Providers in Seven US Cities

**DOI:** 10.1089/apc.2017.0299

**Published:** 2018-07-01

**Authors:** Joanne Simone, Mary Jo Hoyt, Deborah S. Storm, Sarah Finocchario-Kessler

**Affiliations:** ^1^ François-Xavier Bagnoud Center, School of Nursing, Rutgers, The State University of New Jersey, Newark, New Jersey.; ^2^ Department of Family Medicine, University of Kansas Medical Center, Kansas City, Kansas.

**Keywords:** preconception care, HIV, health knowledge, attitudes, practice, health personnel, qualitative research, models of clinical care

## Abstract

Preconception care can improve maternal and infant outcomes by identifying and modifying health risks before pregnancy and reducing unplanned pregnancies. However, information about how preconception care is provided to persons living with HIV (PLWH) is lacking. This study uses qualitative interviews with HIV care providers to describe current models of preconception care and explore factors influencing services. Single, anonymous, telephone interviews were conducted with 92 purposively selected HIV healthcare providers in Atlanta, Baltimore, Houston, Kansas City, Newark, Philadelphia, and San Francisco in 2013–2014. Content analysis and a grounded theory approach were used to analyze data. Participants included 57% physicians with a median of 10 [interquartile range (IQR) = 5–17] years HIV care experience; the mean proportion of female patients was 45%. Participants described Individual Provider (48.9%), Team-based (43.2%), and Referral-only (7.6%) models of preconception care, with 63% incorporating referrals outside their clinics. Thematic analysis identified five key elements influencing the provision of preconception care within and across models: consistency of delivery, knowledge and attitudes, clinic characteristics, coordination of care, and referral accessibility. Described models of preconception care reflect the complexity of our healthcare system. Qualitative analysis offers insights about how HIV clinicians provide preconception care and how key elements influence services. However, additional research about the models and outcomes of preconception care services are needed. To improve preconception care for PLWH, research and quality improvement initiatives must utilize available strengths and tackle existing barriers, identified by our study and others, to define and implement effective models of preconception care services.

## Introduction

Studies have shown high fertility desires and intentions among women and men living with HIV.^1–5^ However, data indicate that a substantial proportion of pregnancies among women living with HIV (WLH),^6^ and among women without HIV infection,^7^ are unintended or unplanned. Preconception care encompasses a spectrum of care that begins with asking patients about their reproductive desires and intentions to assess the need for services that include contraception for those who wish to delay or prevent pregnancy and health promoting counseling and interventions for those who want to have a child.^8,9^ Preconception care is a recommended component of primary^10^ and HIV care^11^ that identifies and addresses biomedical, behavioral, and social risks to a woman's health and pregnancy outcomes before pregnancy as a way to improve maternal and fetal/newborn outcomes and increase planned pregnancies.^12,13^

Preconception care most often focuses on women of reproductive age, but must also address the needs of men. For persons living with HIV (PLWH), goals of preconception care include informing reproductive decision-making and minimizing risks of horizontal and vertical HIV transmission.^14^ Current recommendations for safer conception^11,15,16^ are informed by data showing that PLWH on antiretroviral therapy who maintain an undetectable viral load have effectively no risk of sexually transmitting HIV to HIV-negative partners.^17^ Maternal viral suppression to undetectable levels before conception, during pregnancy, and at the time of delivery makes perinatal HIV transmission almost completely preventable.^18–20^ In addition, a recent study of individuals and couples who were referred for preconception counseling at a large urban HIV clinic found multiple comorbidities and psychosocial issues that may pose increased risks for adverse perinatal outcomes and sexual and perinatal HIV transmission.^14^ These findings emphasize the importance of preconception care for women and men living with HIV.

Despite recommendations from the Centers for Disease Control and Prevention (CDC),^8^ the American Academy of Family Physicians (AAFP),^10^ American College of Obstetricians and Gynecologists (ACOG),^21,22^ and the Public Health Services Panel on Treatment of Women with HIV and Prevention of Perinatal Transmission,^11^ preconception care is not consistently integrated into primary^13,23^ or HIV healthcare^24,25^ in the United States (US). Barriers to these services include inadequate provider knowledge and education about preconception care, lack of confidence in the value of preconception counseling, beliefs that patients will know to seek appropriate care when needed, and concerns about reimbursement coverage for preconception visits.^26–28^ Patient barriers include inadequate knowledge about the need for and benefits of preconception care, discomfort talking with providers about having children, and difficulty accessing preconception care services.^28–31^

Although studies have reported on provider knowledge, attitudes, and practices related to HIV preconception care,^26,28,30,32^ more information is needed for focused efforts to improve services. Steiner et al. discuss strategies for improving implementation of preconception care for PLWH and offer initial recommendations, including considerations about who should provide preconception care, what services should be offered, and where and when they should be provided.^33^ Answers to these questions may guide development of services most feasible in various clinical settings. However, information about existing approaches used to provide preconception care is lacking, and models to optimize the integration of preconception care into primary and specialty healthcare services have not been well-defined or evaluated. The purpose of our study was to address this gap using HIV care providers' accounts of HIV preconception care services in their clinical settings to describe current models used to provide preconception care to PLWH and explore factors influencing preconception care services.

## Methods

HIV healthcare providers' descriptions of preconception care services in their clinics were collected as part of a multisite, mixed-methods study about attitudes and practices related to reproductive counseling, safer conception, and preconception care. Methods have been described previously.^32^ In brief, data were obtained through single phone interviews conducted with a purposively selected sample of 92 healthcare providers from academic and community-based clinical settings in 7 US cities between August 2013 and October 2014: Atlanta (10), Baltimore (14), Houston (13), Kansas City (14), Newark (8), Philadelphia (20), and San Francisco (13). Interview audio recordings were transcribed verbatim and entered into Dedoose, a software program for qualitative analysis.

This analysis focuses on responses to questions about the reproductive health services available for PLWH, including descriptions of how reproductive counseling and services were provided on site or via referral, implementation of services, and institutional support or barriers to providing preconception care. Using a content analysis and grounded theory approach, two of the authors analyzed data to define the construct of interest, models of preconception care services. Data were reviewed to categorize the predominant model described by each participant. Discussion of discrepancies after the initial round of coding led to refinement of definitions and categorizations. Responses were then recoded, with discussion used to reach final consensus. Five key elements and constituent themes that influence services emerged from the analysis. Models and key elements were described within the context of preconception care literature and knowledge about healthcare service delivery in the US. Descriptive analyses were conducted using SAS version 9.4 (SAS Institute, Inc., Cary, NC), Microsoft Excel, and Microsoft Access to characterize participants and summarize described models of care. The study was coordinated by the University of Kansas Medical Center and approved by Institutional Review Boards at the lead site for each city. All participants gave verbal informed consent. Data were anonymous; we did not record or document participants' names or places of employment.

## Results

### Provider characteristics

Participants included physicians (*n* = 52, 57%), nurse practitioners (*n* = 27, 29%), physician assistants (*n* = 6, 7%), nurses (*n* = 3, 3%), and social workers (*n* = 4, 4%) with a median age of 42 years [interquartile range (IQR) = 35–55]. Most were experienced healthcare providers, with a median of 10 [interquartile range (IQR = 5–17] years of experience serving PLWH; 86 of 92 participants had worked with PLWH for at least 2 years. Participants described their areas of specialty practice as HIV (*n* = 44, 48%), infectious disease (*n* = 26, 28%), family practice (*n* = 10, 11%), internal medicine or primary care (*n* = 7, 8%), and women's health or obstetrics and gynecology (*n* = 4, 4%). The majority served patient populations of men and women, with a mean (±SD) proportion of 45% (27.9%) female patients.

### Models of care for providing preconception care services

The models of care and types of referrals used to provide preconception care services are summarized in Table 1. Thirty-four participants (37%) described Individual Provider or Team-based models of preconception care services that were clinic-based and did not use referrals to outside providers, clinics, or facilities. Just over half of participants (*n* = 51, 55.4%) described Individual or Team-based models that included referrals to other providers or clinics within their institution (Internal referrals) or to other facilities (External referrals). Only seven participants (7.6%) described a Referral-only model where care could be provided through referrals if needed. Each model of preconception care is defined in Table 2 with participant quotations illustrating characteristic strengths and limitations for provision of services. We did not identify any quotations exemplifying strengths of the Referral-only model. Participants varied in their abilities to describe preconception care services in their clinics. In part, this variation may be related to models of care, since some participants played a limited role and relied on other team members or clinicians outside their clinic to provide preconception care.

**Table 1.** Models of Preconception Care Services and Type of Referrals Described by HIV Healthcare Providers in Atlanta, Baltimore, Houston, Kansas City, Newark, Philadelphia, and San Francisco, 2013–2014

*Types of models and referrals*	*All* N* = 92* n *(%)*
Individual provider model	9 (9.8)
Individual provider and referral model^a^	36 (39.1)
Team-based model	25 (27.2)
Team-based with referral model^a^	15 (16)
Referral-only model^a^	7 (7.6)
Referrals to providers/clinics within the health system (internal referrals)	31 (33.7)
Referrals to providers/clinics outside the health system (external referrals)	22 (23.9)
Internal and external referrals	5 (5.4)
No referrals	34 (37)

^a^ Referrals to other providers or clinics within their institution (internal referrals) or to other facilities (external referrals).

**Table 2.** Models of HIV Preconception Care Services with Characteristic Strengths and Limitations Illustrated by Quotes from Providers in Atlanta, Baltimore, Houston, Kansas City, Newark, Philadelphia, and San Francisco, 2013–2014

*Defined models*	*Descriptions and examples of model strengths*	*Descriptions and examples of model limitations*
Individual provider model An individual healthcare provider provides the majority of preconception care to the patient.	Convenient for the patient. May influence other providers or clinic culture by being a champion of preconception care. • *I provide comprehensive reproductive health services…I can offer pretty much everything. From family planning, to pre-conception counseling, to surgery if indicated.*	Provider dependent, not necessarily a clinic-wide standard. Quality may vary by provider and patients may receive inconsistent preconception care. May not be consistently available or sustainable if provider-dependent and not an expectation of all providers. • *So…you know, depending upon the provider….we don't have a specific program set up to say… this is your prenatal counseling….So is it adequate, probably not, we probably need to do a little bit more aggressive… intervention early to make sure they… understand about viral load and risks and all that stuff.*
Team-based care model Provision of preconception care is shared between healthcare providers within a clinic.	Care is convenient for the patient. Although a separate appointment may be necessary to see a particular member of the team, the model is more sustainable as the clinic culture incorporates preconception care as a clinic-wide standard. • *Family planning, preconception planning, colposcopy services, GYN services, and sexual health services. They're available 5 days a week during all clinic hours, so that's 8 h a day, 5 days a week.*	Model may be resource dependent. In-depth counseling or services dependent on availability of point person. A system of communication between providers must be in place for optimal coordination of care. • *We have a nurse, I believe, that's available to help counsel patients…who want the counseling. I'm not sure what…the services are at our location to my knowledge.*
Individual or team-based care with referral Initial assessment of need is done by the provider or team but a referral outside of the clinic is given for more comprehensive counseling or for services not available within the clinic setting.	Sustainable model. • *We, in our clinic, can provide some counseling about the different conception options and about very effective preconception care and then we have very easy access to [a referral organization] for more extensive counseling and preconception consults for HIV positive women for HIV negative women who have HIV positive partners. So, we're very lucky to have access to those services and that expertise.*	Inconvenient for the patient (making another appointment and traveling to appointment site). If referral is outside the system, sharing of medical records and coordination of care between providers may be more difficult. Provider must be familiar with the referral site and their level of HIV expertise. • *If the patient is seeing us for primary care, I'm not sure how that would work for referring them to this other practitioner. It might have to be for the counseling, her just giving me the information, and me giving it to the patient.* • *Now, as to… how well they [referred provider] do the counseling, I don't know. But I assume that that is part of what they do…*
Referral only Preconception care is not an integral part of services, but referrals are made in response to patient requests or specific needs.	None	Requires patient knowledge and awareness of preconception care needs and ability to request specific services. Because preconception care is not considered part of services, knowledge of referral options may be limited. Inconvenient for the patient (see limitations of “Individual or Team-Based Care with Referral” model.) • *…we have a…reproductive…a couple reproductive specialist and I mean I'd send them to those folks. Again, it hasn't arisen yet…* • *I would say similarly with the females, it's not my area of expertise. I think, especially for some of the assisted reproductive technology questions, it's been difficult to know where I can… refer clients without insurance…. I think that there's a lot of questions there.*

### Key elements of preconception care services

Participants discussed issues reflecting five key elements that influenced the provision of preconception care services: consistency of delivery, provider knowledge and attitudes, clinic characteristics, coordination of care, and referral accessibility. Participant quotations illustrating themes within the key elements are listed in Table 3 and depict how each element can support or pose barriers to preconception care services. We noted that key element themes varied within and across models. Some participants discussed high-functioning delivery of preconception care services, while others described the same model of care, but with less effective service delivery due to lack of provider knowledge, difficulties with referrals, and so on. The following sections explore how each key element affects preconception care.

**Table 3.** Key Elements and Themes Influencing Preconception Care Services: Exemplary Quotes from HIV Healthcare Providers in Atlanta, Baltimore, Houston, Kansas City, Newark, Philadelphia, and San Francisco, 2013–2014

*Key element*	*Quotes from providers*	*Themes influencing service delivery*
Consistency of delivery	• *If I had a female patient who was still able to have…children and they are, from a health perspective, at a stage in their health where we can start discussing future…quality of life or social questions that they may have including pregnancy in the future…* • *Really for both men and women, lots of our HIV-infected folks really want to have children, so that's really a part of the initial subject areas…*	Population-specific care as determined by the provider
	• *I feel like affected individuals are probably not proactively queried about their reproductive desires.* • *If they ask me, I will talk with them about pre-conceptual counseling and I've done that with several of my patients.*	Patient-driven rather than provider-driven
Provider knowledge and attitudes	• *I think because we have access to this woman who has a lot of experience counseling discordant couples, she's given talks to us, I feel pretty comfortable or confident that I can give them accurate information.* • *I feel fairly comfortable with my experience.… So I feel comfortable in starting the conversation in what a woman's choices are, does she want to conceive, does she not want to conceive.*	Confidence to discuss
	• *…I feel I do not have the expertise to give them all the options that they might have… in terms of HIV risk and what we can do…* • *Family practitioners such as myself do…oral contraceptives and…regular woman's healthcare. But we don't have any fertility specialists here.* • *I don't have enough of a knowledge base for the male patients regarding sperm washing… I'm aware that there is someone in the city…. So I certainly would refer a male patient who was seeking answers more than what I had to give, I would refer them out.*	Lack of specific knowledge or training
Clinical characteristics	• *…We all feel pretty strongly that … our HIV patients should be able to have families…. I think we all feel like we need to get better at…helping people do that and talking about it.*	Culture of support for PCC
	• *I would say that it would be helpful to have more structured kind of protocol around it which is something that we're actually really working on now. We're rolling out our electronic medical records and really kind of shifting some of the more free form work that we've been doing into actual more checking off lists and making sure at every visit that particular things are addressed.* • *In terms of counseling….That is available, the expertise is here. We have a protocol in place…*	Clinic level supportive tools and resources
	• *I don't think I'm encouraged to address reproductive plans and I don't think I've received training.* • *…we don't have like a formal…we don't have like a group even, we don't have one particular person who does reproductive counseling with HIV positive female… we don't have trainings on them.*	Lack of institutional support for PCC
Coordination of care	• *We try to…plan for the next patient's visit be it this week or in two or three weeks so we…will have those discussions…male patient is desiring to conceive… or a female patient. So we do have the multidisciplinary…meeting with our case managers, behavioral health services, peer, adherence counselors and other folks at that visit.* • *…we liaise directly with obstetric services, and there are counselors and providers in the main hospital where I work.* • *I have referred a number of people to [OB/GYN]…so if there's questions we just kind of ask each other…that is actually something [that] comes up in provider meetings…*	Within system coordination
	• *What I do is if a patient needs a referral, [my nurse] and I…work side by side and so if there's a patient that needs to have a referral…she'll usually contact the agency or the institution where the patient is going to be referred to fax over the information….we usually have the patients come back in two weeks to make sure that they have actually had a consult that they've been contacted. If not, then we'll usually call back the agency ourselves to make sure to see whether the patient is on the list.*	External coordination
	• *We do have an education department at our clinic, but I'm not sure they do a whole lot of prenatal counseling. And that's just ignorance on my part, they very well may, I just don't know if they do.* • *…I feel like I'm not well aware of the resources in the community.… I wish I just had more providers to talk with, even to consult on the side to see what kind of approaches that would be.*	Limited awareness/coordination
Referral accessibility	• *…we have a whole range of reproductive health providers who care for HIV-infected women. And they're quite readily available in the building within which we work.* • *We actually have a women's health nurse practitioner who we refer clients to for issues around reproductive health. She sees them both in this clinic and she can also see them in OB/GYN clinic as well, but primarily she sees them in our clinic.* • *…I'm not aware of anything specific. I think again as a general referral I could refer the patients, but we don't have anything…as physicians treating a group of HIV infected patients, we don't have any onsite service available, that would depend on the OB/GYN department.*	Referral availability
	• *They are available by referral, but they are not easily available. Trying to get an appointment to make the appointment there's so much time lapsed that people think they'll not make the appointment.* • *For a long time, they were waiting, like, months where nobody would pick up the phone. They would be told that they'd get an appointment at some point. And I finally ended up paging, like, the residents, and then they told me that there's a secret walk-in clinic, and that they can walk into that.* • *…honestly I cannot tell you the details, my social workers handle that referral process. But the patients do have to acquire a gold card, which is their access mechanism…*	Referral accessibility

OB/GYN, obstetrics and gynecology; PCC, preconception care.

#### Consistency of delivery

Consistency of delivery refers to the extent to which preconception care services were described as a regular component of comprehensive HIV care. Participants gave insights about how preconception care was delivered in their clinical settings and who they saw as priority populations for these services. Some discussed engaging all patients in conversations about reproductive plans, while others talked about choosing patients for these conversations, that is, triaging patients based on gender, sexual orientation, health, socioeconomic, or relationship status. The frequency with which providers engaged in conversations about reproductive goals ranged from a routine component of each visit, to episodically, or only in response to a patient question or request.

#### Provider knowledge and attitudes

To integrate preconception care services within HIV clinics, providers need core knowledge that enables them to assess reproductive intentions on a regular basis, incorporate defined content into counseling, and provide or refer patients for needed services. Some participants were knowledgeable about the content and provision of preconception care, while others had difficulty describing these services because they had limited understanding and experience providing them. Some participants referred to other reproductive health services, such as routine gynecological care and cervical cancer screening, rather than preconception care. A number of providers described a lack of training in this area and/or discomfort with the subject matter which posed a barrier to providing services. Access to training and advice from an expert can increase comfort and confidence in providing preconception care, particularly for serodifferent couples. This is important because knowledge about preconception care can positively influence provider attitudes regarding the importance of preconception care, perceived patient benefits, and belief in that it is within their scope of practice, and willingness to provide services.

#### Clinic characteristics

The clinical setting and healthcare system can positively or negatively affect providers' abilities to incorporate preconception care into HIV services. Shared attitudes about the importance and relevance of preconception care services support integration. Absence of clinic level supports and lack of an environment that fosters reproductive health discussions were noted as detrimental to preconception care services. Participants did identify available and desired supportive resources for the delivery of preconception care such as expert clinician champions within the clinic setting, support for training, electronic medical record prompts, clinician tools and resources, and/or policies and procedures.

#### Coordination of care

Models of preconception care described by most participants require coordination of services with other team members and/or providers accessed through referrals. Coordination of care refers to the deliberate organization of patient care activities with information sharing to support effective care.^34^ Clinicians need to understand and communicate about their respective role in providing services. Knowledge about the plan of care is needed to assist patients in following the treatment plan. In stronger descriptions of care coordination with providers outside their clinics, participants emphasized the importance of patient follow-up. Others seemed to be missing necessary components of effective care coordination; participants lacked knowledge about the type of care the patient would receive through referrals or did not mention communicating about the plan of care.

#### Referral accessibility

Participants who described working in functional systems could easily identify the types of preconception care services that required referral, depending on the structure and resources of the clinic. For example, some participants preferred to refer patients to a clinician specializing in safer conception counseling, while others could perform this service themselves. In addition, the availability of services for contraception varied among clinics. Once providers identified areas where they needed additional expertise, their knowledge of referral resources was integral to enabling patient access to those services. Long wait times, transportation issues, insurance barriers, or difficulty making appointments were described as barriers to patients' abilities to receive services.

## Discussion

Our qualitative study about reproductive counseling enabled us to use clinician perspectives to describe current models of preconception care, increasing our knowledge and understanding about how these services are provided to PLWH. Almost all the 92 HIV clinicians interviewed described using an individual provider or team-based model of preconception care services, with about half of them also referring patients to providers outside their clinic (but within their institution) or to other organizations and agencies. The few participants describing a Referral-only model did not provide preconception care as part of HIV care, but indicated they would refer patients in response to a request or specific need. Importantly, we identified five key elements with common themes of positive and negative influences on the provision of preconception care services. Key elements and themes were not specific to a particular model but varied within and across models of care.

A 2006 review pointed out that despite increasing knowledge about how preconception care can improve pregnancy outcomes, there is limited information about how this information is translated into practice.^35^ Findings of a 2016 systematic review suggest that the situation has not changed substantially and concluded that research has not focused sufficiently on the clinical reality of preconception care services.^28^ Steiner et al. proposed studying two general models: (1) preconception care as an integral part of comprehensive HIV care and (2) linkage and referral to preconception care services.^33^ Our findings suggest a third model where referrals are used to supplement clinic-based services by individual HIV care providers or teams. These approaches align with described US HIV care delivery models: (1) individual provider models for physicians and advanced practitioners, (2) team-based models of colocated multidisciplinary services, and (3) shared care that involves referrals and comanagement with other providers outside the HIV clinic or practice setting.^36^

Preconception care is recommended as an integral, ongoing part of primary care^10^ and the care of PLWH.^11^ However, preconception care is often missing from primary care of adults,^23^ and it is not yet a standard component of HIV care,^4,24,37^ although PLWH may receive primary care in HIV clinic settings.^38–40^ When reproductive conversations do occur, they may often emphasize contraception and prevention of HIV transmission through condom use, rather than exploring the patient's reproductive desires or plans.^26^ A 2013–2014 Medical Monitoring Project survey of 1234 providers found that only 49% provided comprehensive reproductive health counseling, although individual components were addressed more often, for example, assess reproductive intentions (71%), explain perinatal HIV transmission risk (78%), provide contraception (76%), and refer for preconception care (64%).^24^

Exploration of participant quotations associated with key element themes provides information about how each element can affect services. Federal guidelines recommend preconception care services for women and men.^8^ However, consistency of delivery is affected by providers' knowledge and standards about who should receive preconception care. We found that some providers described limiting conversations about reproductive goals and preconception care to certain patient populations, such as women of childbearing age or women they perceived as ready for pregnancy. A high proportion of men, or gay men, in a provider's practice were cited as another reason why preconception care was not a part of their services. These observations highlight the need for practice standards that align with national guidelines. Consistency of delivery is also impacted by whether services are driven by the provider or by the patient. Although some providers were proactive, others described responding to patient needs or requests. This is problematic, since patients may be reluctant to raise questions or express their needs, and they may be unaware of the importance of preconception care.^26,29,41^ The persistent legacy of stigma against childbearing by WLH may reduce both patients' and providers' willingness to initiate discussions about reproductive desires and plans.^42^

Participants frequently perceived the need for referral to reproductive specialists to conduct preconception care counseling due to knowledge deficits. This is consistent with other studies that found provider knowledge deficits about safer conception strategies, particularly for serodifferent couples.^16,26,28^ Yet, safer conception interventions, such as self-insemination, timed condomless intercourse, and HIV preexposure prophylaxis (PrEP) can be provided by HIV clinicians with developed expertise.^11^ Consultation with an obstetrician or assisted reproductive technologies may be indicated for some patients, the misperception that they are required for patients to conceive safely can become a barrier to patients receiving adequate preconception counseling. In an earlier analysis from this study, only 19% of participants had experience prescribing HIV PrEP.^32^ A recent qualitative study with couples using PrEP for safer conception found that patients value the ability to conceive naturally through condomless sex.^43^ Findings from our study and others emphasize the need for provider education about safer conception options.

The majority of participants described models of preconception care that require them to work with others—within clinic teams and/or with referral providers. It is important to acknowledge and address practice barriers, including time constraints and competing priorities in clinic visits, through enabling resources.^27^ Clinic characteristics, such as assessment tools, protocols, and educational resources can help providers identify and address patients' needs.^27^ Clinics can also define components of preconception care that should be addressed by HIV providers and develop referral resources and procedures for coordinating care that is beyond their defined scope of services.^24,33^ Because the use of referrals increases the complexity of care and can affect patients' abilities to obtain services, clear communication with coordination of care and evaluation of referral accessibility are particularly important.

Some of our study's limitations have been discussed previously and include potential selection bias and social desirability of responses based on interest in or expectations about preconception care services and variations among interviewers.^32^ The study was strengthened by the inclusion of varying types of HIV clinicians in community- and academic-based settings in seven cities that reflect the diversity of Mid-Atlantic, Southern, Midwest, and West regions. It was beyond the scope of this study to assess clinic structure, staffing, and overall model of care in the HIV clinics where participating clinicians worked. Each principal investigator has expertise in perinatal HIV services. Therefore, our findings may not be representative of cities without perinatal HIV programs or rural areas. Furthermore, interview questions were not designed to elicit details that would have allowed meaningful comparisons between models of care. Preconception care was not specifically defined as part of the interview, so clinicians' responses may be based on differing levels of understanding or differing perceptions of what was most important to share. For these reasons, although we noted that key elements reflecting stronger preconception care services occurred across models, we did not try to quantify these findings or make statistical comparisons.

Study participants described not only strengths of preconception care services but also pointed out existing weaknesses. Multiple organizations have called for improvement in preconception care.^12,23^ Advancing preconception care for PLWH begins with defining it as core component of HIV care and assuring that clinicians have adequate knowledge and resources to provide services. The CDC and US Office of Population Affairs,^8,44,45^ ACOG,^21,22^ and the Public Health Services Panel on Treatment of Pregnant WLH, and Prevention of Perinatal HIV Transmission^11^ recommend specific content areas of counseling and services. Because preconception care requires ongoing care and interventions that are tied to health promotion, HIV management, and prevention, HIV care providers are well positioned to play a lead role through direct services and referrals.^24^ However, support for implementation is essential, as exemplified by factors affecting implementation of chronic care models: appropriate resources, acceptability of the intervention to providers and patients, provider preparation, and patient support.^46^

Available data indicate that systematic efforts are needed to strengthen preconception care in the United States. Described models of preconception care services reflect the complexity of our healthcare system. Quality measures for core content components of preconception care have been identified,^47,48^ but quality measures for the process of service delivery are also needed. Our findings can be used to inform future health systems research about models of care and strategies to improve the delivery and outcomes of preconception care services. The key elements we identified could be further developed and operationalized for use within the context of research or continuous quality improvement. Examples of strategies to strengthen preconception care services, organized by the key elements, are summarized in Table 4. These strategies are derived from existing literature, national guidelines and recommendations, and the expertise of practicing clinicians and researchers shared through formal and informal national meetings and presentations. Clinicians and clinical programs can identify how their system functions and which of the elements they should prioritize to improve existing services. To improve preconception care for PLWH, future research and quality improvement initiatives must utilize available strengths and tackle existing barriers, identified through our study and by others, to define and implement effective models of preconception care services.

**Table 4.** Examples of Strategies to Strengthen Preconception Care Services, Organized by the Five Key Elements

*Key elements influencing preconception care services*	*Examples of strategies to strengthen preconception care services^a^*
Consistency of delivery	Define core, minimum care for entire patient population.
	Integrate into routine care.
	Utilize electronic medical record prompts and reminders.
	Utilize standardized questionnaires that include questions about reproductive intentions.
Knowledge and attitudes	Identify or assess training needs: Integrate questions about provision of preconception care into provider and patient needs assessments.
	Offer regular training to providers. Provide different types of training opportunities for example, didactic, case study, preceptorship/mentor training, etc.
	Integrate preconception care content or focus into other trainings about health maintenance, that is, well patient care for HIV primary care or preexposure prophylaxis.
Clinic characteristics	Define staff member roles and responsibilities in the provision of preconception care.
	Engage expert clinician champions within the clinic setting.
	Set clinic wide expectations around preconception care services, incorporate in policies and procedures.
	Make preconception care resources visible in clinic spaces.
	Support training opportunities.
	Integrate EMR prompts and other support technologies.
	Determine team roles and organization workflow around preconception care tasks.
	Provide patient education about reproductive health as a part of HIV care.
	Create a quality improvement process around the provision of preconception care that is, chart review and Plan, Do, Study and Act (PDSA) cycle.
Coordination of care	Create policies and procedures for assisting patients in scheduling appointments for referrals, following up on patient appointments for referrals, staff responsibilities for ensuring transfer of medical records and/or other documentation, and provider communication before and following the referral, that is, communicating goals for the appointment and plan of care.
	Investigate and implement EMR and other technologies to assist in implementing the policies.
	Utilize all staff members to their highest level to facilitate smooth coordination and communication.
Referral accessibility	Create an accessible list of referral points and contact information for all anticipated areas of care that is, contraceptive access, assisted reproductive technologies, OB/GYN, etc.
	Define responsibilities for assisting patients with barriers to accessing care at referral appointments.

^a^ These strategies were derived from descriptions provided by participants, existing preconception care literature, national guidelines and recommendations, and the expertise of practicing clinicians and researchers shared through formal and informal national meetings and presentations.

EMR, electronic medical record; OB/GYN, obstetrics and gynecology.
